# Directions for Using the Lunar Caustic

**Published:** 1827-04

**Authors:** John Higginbottom

**Affiliations:** Member of the Royal College of Surgeons of London.


### LUNAR CAUSTIC.

*Directions for using the Lunar Caustic.* By JOHN HIGGINBOTTOM, Esq. Member of the Royal College of Surgeons of London. (Communicated by Dr. MARSHALL HALL.)

I AM desirous of giving a distinct account of the plan which I have learnt from experience to be the best, in applying the lunar caustic in those diseases in which I have hitherto employed it; for the *proper mode* of application of the caustic is quite essential to secure its good effects, and to avoid some rather disagreeable consequences of a careless mode of using it.

In the first place, I always prefer to use the lunar caustic in its solid form; for it is in that state much more manageable than in any other. It is necessary to moisten the surface to which it is applied slightly with pure water, except in the case of ulcers, from which lymph or pus exudes, and then this is only necessary in regard to the surrounding skin.

In the second place, it is essential to know the precise effects of the lunar caustic, in the different degrees of its application. If the caustic be passed once slightly over the moistened skin of any part, except the hand (upon which the cuticle is thicker than elsewhere,) it induces an eschar simply; if it be passed over the surface twice or thrice, to the eschar will be added some vesication; if more frequently still, there will be vesication only. In the first case, there will be no pain; in the second and last, there will be soreness proportionate to the degree of vesication.

It is essential to the success of this plan of treatment by the lunar caustic, that these observations be kept constantly in view.

I shall now first describe the mode of application of the caustic in the treatment—

1. *Of recent bruised wounds of the shin, &c.*—In recent bruised wounds of the shin, the caustic should be applied upon the wound, taking care to leave no spot untouched, and upon the surrounding skin to the breadth of one-third of an inch, in such a manner as to induce an eschar without vesications. Any moisture which may remain upon the wound is then to be removed, by gently applying a little linen or lint, and the skin surrounding that to which the caustic was applied is to be moistened, and covered with goldbeater's skin, so that the whole may be protected from accident; the parts are then to be kept cool, free from covering, and exposed to the air.

This is usually all the treatment which is required in this kind of injury. I have generally found that an adherent eschar is formed, and that no further application or attention is required, except in old people, in whom the skin is sometimes irritable from various causes: in this case a little fluid will form upon the edges of the eschar, and will require to be evacuated by a small puncture, as in the treatment of ulcers about to be described; the goldbeater's skin being removed for this purpose, and then reapplied.

If the eschar be removed by accident at any time, the application of the caustic must be repeated as before. If due care be taken to avoid this kind of accident, I have not, in general, found it necessary to enjoin rest.

2. *Of small ulcers.*—I have stated, in my Essay on the Application of the Lunar Caustic, what were the cases in which I supposed it was proper to use this remedy. I have, since the date of that publication, improved much upon the mode of its application, and discovered many new instances of its utility.

The treatment of ulcers by the caustic, certainly requires more care and attention than some other cases; yet I have seldom found it necessary to attend daily to them for more than nine or ten days. It is of the greatest importance that the application of the caustic should be made with the utmost care; I shall, therefore, be very explicit in giving my directions for this purpose.

The surrounding skin is first to be moistened, and the caustic applied lightly, so as not to induce vesication, to the extent of half an inch round the ulcer. It is then to be applied over the ulcerated surface; and it may be applied more freely upon this surface than in the case of a recent wound. The whole is then to be protected by goldbeater's skin, in the manner already described.

The application of the caustic round the ulcer subdues the inflammation of this part, and induces a firmer, and more continuous; and adherent eschar. If any detached vesication be induced, it is to be simply exposed to the air; but if it communicate with the surface of the ulcer, the fluid is to be carefully evacuated. A light dress, as wide trowsers, if the seat of the ulcer be upon the leg, is to be worn.

On the succeeding day, the goldbeater's skin is to be removed, by being moistened with a little water: a small smooth incision is to be made, by means of a penknife, through the eschar in its central part, and then a little pressure is to be made, so as to evacuate any fluid which may have been effused; this fluid is to be carefully removed by a little soft linen; the breach in the eschar is to be repaired by reapplying the caustic; and the whole is to be protected, as before, by the goldbeater's skin.

On the first and second days, there is usually little fluid secreted; for five or six succeeding days, rather more is formed. The same means must be employed for evacuating the fluid every day, until the eschar finally becomes completely adherent. This will be ascertained by the appearance of indentations in the surface of the eschar, and usually occurs about the tenth day. It is remarkable that, in cases in which an eschar has been formed over a slough, it has required double the number of days to become adherent.

During the unadherent state of the eschar, it is proper to administer an efficient purgative medicine every second or third day, and to enjoin rest. Afterwards it is necessary carefully to remove the portions of the eschar as they separate at the edges, by means of a sharp pair of scissors, and to take great care to preserve it in its situation by the goldbeater's skin, and from being detached by accident.

3. *Of punctured wounds and bites.*—In *recent* punctured wounds, the orifice of the wound must be first examined: if there be any loose portion of skin closing the orifice of the wound, it is to be removed by a pair of sharp-pointed scissors or by a lancet; the puncture, and the surrounding skin, are then to be moistened with a little water; the caustic is to be applied to the former until some pain be experienced, and over the latter lightly, so as not to induce vesication. The caustic is then to be applied to the skin, for an inch round the puncture, and to a greater extent if the swelling exceeds this space. The part is then to be exposed to the air.

These cases are generally adherent from the first application of the caustic, but I have sometimes found the eschar to separate from the wound before it has healed, owing to its conical form: it is then only necessary to repeat the application of the caustic slightly, to complete the cure.

At a later period of punctured wounds, inflammation is usually present, the punctured orifice is nearly closed by the swelling, and a little pus has generally formed within. A slight pressure is to be applied to evacuate this fluid; the caustic is then to be applied within the puncture, and upon and a little beyond the surrounding inflamed skin, and the parts are to be exposed to dry. In this manner an adherent eschar is formed, and the inflammation subsides. If there be any vesication, it may be simply left to nature; the fluid is soon absorbed or evaporates.

If there be reason to suppose that an abscess has formed deeply, it must be opened freely by the lancet, and the caustic is then to be applied within the cavity; a poultice of bread and water, and cold water as a lotion, are then to be applied over the whole. The application of the caustic may be repeated every second or third day, if the swelling or inflammation require it; and the cold poultice may be renewed every eight hours.

I have several times applied the caustic over an inflamed surface, in cases in which I was not aware that suppuration had taken place. Even in these instances, an immediate check was given to the surrounding inflammation, and relief to the pain; but, two or three days afterwards, there was an increase of swelling, attended by some pain, which is not usual except when there is matter or some extraneous body underneath. In these cases I made a free incision with the lancet, and applied the caustic and cold poultice.

4. *Of external inflammation.*—I have had many opportunities of trying the efficacy of the lunar caustic in the treatment of external inflammation, and have published some examples of this mode of cure in this Journal for May and June, 1826.

In this case it is best, first to wash the part with soap and water, to remove any oily substance from the skin, and to wipe it dry; then to moisten the inflamed and surrounding skin, and to apply a long stick of caustic flat upon the moistened surfaces, taking care that not only every part of the inflamed skin be touched, but the surrounding healthy skin, to the extent of an inch or more. The caustic must be passed over the surface twice or thrice only. The part is then to be exposed to the air to dry, and to be kept cool.

In twenty-four hours, if the caustic has been properly applied, it will be observed that the inflammation has greatly subsided, and its progress been checked; but, if there be one spot left untouched, the patient complains of it. Every such spot must be touched with the caustic. At this period there is usually a little vesication, which, however, only does good, and never increases the inflammation or induces irritation.

On the third day, there is usually more vesication and less swelling, and the patient complains of a little pain, as of that of a blister; but, on pressure, the part has a puffy feeling, and is quite free from inflammation.

On the fourth day, the vesications are disappearing. It is best to leave them undisturbed, for the dried exudation defends the subjacent cutis.

On the fifth day, the vesicated crusts separate, leaving the subjacent parts free from soreness or inflammation. It is sometimes several days before the whole of these crusts peel off; but I believe it is best to leave them undisturbed.

In *erysipelas from wounds or ulcers,* the wound or ulcer, and the inflamed surface, are to be treated by combining these modes of using the caustic.

In *inflammation of the absorbents,* the caustic is to be applied as in external inflammation, passing it along the course of the inflamed absorbents, and beyond the inflamed surface in every direction.

5. *Of constitutional erysipelas.*—In this affection, bleeding, emetics, and purgative medicines are to be premised, and then the lunar caustic is to be applied in the following manner:—The caustic is to be applied over the whole inflamed surface, and beyond it upon the surrounding skin, to a far greater extent than in phlegmon,—perhaps to the extent of two inches or more round the inflamed border of the erysipelas. Any fresh accession of erysipelas must be immediately treated in the same manner. By means of the caustic, I believe it will often be found that we have a complete control over this disease. If the erysipelas be attended by vesication, the vesicles should be broken, and the part touched with the caustic; but, if vesications arise from the use of the caustic, they may be allowed to remain undisturbed. When the erysipelas has affected the head, the scalp should be shaved, that there may be no impediment to the due application of the remedy.

6. *Of phagedenic ulcers.*—In phagedenic ulcers, the caustic is to be lightly applied to the whole ulcer, but particularly to its edges and over the surrounding skin. If the ulcer be situated on the glans penis, a little lint is to be left upon it; if on any other part, the cold poultice and lotion are to be applied.

7. *Of the pain from applying the lunar caustic.*—I have never found the pain induced by the application of the caustic any barrier to its use. Patients generally suffer infinitely more from the inflammation, wound, or ulcer, treated in the ordinary way. The caustic gives a little pain at the time, but this is soon over. The ordinary mode of treatment is both more troublesome and painful, and for a much longer period. From the application of the caustic in some painful circumstances, the patient experiences early, if not immediate relief; and perhaps sleeps for the first time, after passing many restless nights.

I have never observed the least bad consequences from the proper use of the caustic, though this, like all other remedies of great efficacy, requires to be employed with a due attention to such rules as experience teaches us to be best adapted to secure the objects which we have in view.

*Nottingham; March* 1827.

